# A taxonomy of threat and soothing influences in rheumatic and musculoskeletal diseases and central sensitivity syndromes

**DOI:** 10.1016/j.ijchp.2023.100420

**Published:** 2023-11-23

**Authors:** Kim Hijne, Lotte Gerritsen, Ana M. Pinto, José A.P. da Silva, Jonna F. van Eck van der Sluijs, Rinie Geenen

**Affiliations:** a Altrecht Psychosomatic Medicine Eikenboom, Zeist, the Netherlands; b Department of Psychology, Utrecht University, the Netherlands; c Center for Research in Neuropsychology and Cognitive and Behavioral Intervention (CINEICC), Faculty of Psychology and Educational Sciences, University of Coimbra, Portugal; d Faculty of Medicine, University Clinic of Rheumatology, University of Coimbra, Portugal; e Psychological Medicine Institute, Faculty of Medicine, University of Coimbra, Portugal; f Coimbra Institute for Clinical and Biomedical Research (i.CBR), Faculty of Medicine, University of Coimbra, Portugal; g Rheumatology Department, Coimbra University Hospital, Portugal; h Mentaal Beter, Utrecht, the Netherlands

**Keywords:** Affect regulation, Central sensitivity syndromes, Concept mapping, Rheumatic and musculoskeletal diseases, Personalized treatment

## Abstract

**Background:**

An imbalance in affect regulation, reflected by a hyperactive threat system and hypoactive soothing system, may impact physical symptoms in people with rheumatic and musculoskeletal diseases (RMD) and central sensitivity syndromes (CSS), including chronic fatigue syndrome, fibromyalgia, and irritable bowel syndrome. This study aimed to identify and structure comprehensive overviews of threat and soothing influences that may worsen or alleviate physical symptoms in people with RMD or CSS.

**Method:**

A concept mapping procedure was used. An online open-question survey (*N* = 686, 641 [93.4%] women) yielded comprehensive sets of 40 threats and 40 soothers that were individually sorted by people with RMD or CSS (*N* = 115, 112 [97.4%] women).

**Results:**

Hierarchical cluster analyses generated eight threat clusters: environmental stimuli, physical symptoms, food and drugs, inactivity, demands, effort, invalidation, and emotional stress. Ten soother clusters were identified: social emotional support, rest and balance, pleasant surroundings, illness understanding, positive mindset and autonomy, spirituality, leisure activity, wellness, treatment and care, and nutrition and treats.

**Conclusions:**

Our study provided a comprehensive taxonomy of threats and soothers in people with RMD or CSS. The results can be used in experimental research to label threat and soothing stimuli and in clinical practice to screen and monitor relevant treatment targets.

## Introduction

Physical symptoms such as pain and fatigue as well as associated functional impairments represent a major burden in individuals with rheumatic and musculoskeletal diseases (RMD) and central sensitivity syndromes (CSS). RMD encompass more than 200 diseases, including osteoarthritis and inflammatory rheumatic diseases, such as rheumatoid arthritis. The label CSS has been used to refer to chronic fatigue syndrome, fibromyalgia, and irritable bowel syndrome among other syndromes (Yunus, 2008). While inflammatory activity and tissue damage are considered to play a prominent role in RMD and central sensitization is presumed to be the predominant pathophysiological mechanism in CSS (e.g., Minhas & Clauw, 2021; Yunus, 2008), it is generally believed that biological, psychological, and social factors play a role in the onset, development, and persistence of both RMD and CSS (Geenen et al., 2018; Meeus & Nijs, 2007; Tanaka et al., 2011). However, it is assumed that the strength and directionality of specific factors involved in the modulation of physical symptoms differ across and within conditions and individuals (Gavilán-Carrera et al., 2022).

Neuropsychological theories posit that information-processing structures (e.g., salience networks, schemas, mental representations, neuroception) subconsciously prime specific behaviors, cognitions, and emotions. Gilbert (2009, 2014) emphasized such a role for the threat and soothing affect regulation systems. The threat system is programmed to detect and evaluate impending or anticipated threats and promote automatic defensive actions, while the soothing system is linked to a sense of safeness, exploration, and affiliative behaviors as well as positive affect states (Gilbert, 2009). It has been proposed that an imbalance in the affect regulation systems, reflected by a hyperactive threat system and a hypoactive soothing system, may be a diathesis and maintenance factor for physical symptoms in fibromyalgia (Pinto et al., 2023). Although the precise neurophysiological mechanisms will differ across illnesses and individuals, we assume that the threat-soothing balance model can also be used to understand modulation of physical symptoms in other CSS besides fibromyalgia as well as in RMD. The type and number of threat- and soothing-related behaviors, cognitions, and emotions triggered by these systems are extensive and vary among persons. To be able to examine these threat and soothing influences and their potential role as a modulator of physical symptoms, an extensive overview is needed. In treatment, this overview may be helpful to monitor and target individual threats and soothers. In experimental research, it may be useful to identify and rate the threat and soothing valence of stimuli.

Multiple review studies have outlined factors associated with physical symptoms in RMD and CSS, such as infections, psychological symptoms (e.g., depression, anxiety, posttraumatic stress), sleep problems, maladaptive cognitive-affective processes (e.g., catastrophizing, alexithymia), social processes (stigmatization, social support), and coping responses, such as acceptance and self-efficacy (e.g., Adams & Turk, 2015; Geenen & Dures, 2019; Minhas & Clauw, 2021; Monden et al., 2022; Van Middendorp & Evers, 2016). Questionnaires have been developed to measure constructs related to threat, for example, maladaptive schemas (Oei & Baranoff, 2007; Schmidt et al., 1995), and soothing, for example, social safeness (Gilbert et al., 2009), psychological safety (Morton et al., 2022), and self-compassion (Neff, 2003). One questionnaire specifically assessed emotional climate in residential care for youth according to three affect regulation systems: threat, soothing, and drive (Santos et al., 2023). Commonly, these questionnaires are based on input of professional experts rather than patient input as a source of information, show only a few general (latent) factors derived from correlation patterns in groups instead of a comprehensive overview of multiple factors pertaining to individuals, do not assess influences that are related to physical symptoms, or do not make a distinction based on threats and soothers as guided by Gilbert's (2009, 2014) model of affect regulation. Our study adds knowledge to these studies by (1) focusing on threat and soothing influences on physical symptoms guided by Gilbert's (2009) theory, (2) aiming at a comprehensive taxonomy of influences that are essential to an individual, and (3) using the input of people with physical symptoms as a source of information. Our aim was to identify and structure comprehensive overviews of threat and soothing influences that may worsen or alleviate physical symptoms in individuals with RMD or CSS.

## Methods

A concept mapping procedure was used (Trochim, 1989). This method of generating and hierarchically structuring opinions from the perspective of experts by experience is considered valid and reliable (Rosas & Kane, 2012). Using this method, participants individually sort items in a card sorting task after which a statistical technique hierarchically structures these sortings. Items are derived from interviews or open survey questions. Our data were collected and analyzed in two consecutive studies. The aim of the online survey of Study 1 was to acquire a comprehensive set of threats and soothers to be able to identify and select representative sets of threats and soothers. The selected threat and soothing items were printed on cards that were individually sorted by the participants in Study 2 and later grouped by means of hierarchical cluster analysis.

## Study 1: online survey

### Participants

Eligible participants were aged eighteen years or older with chronic physical symptoms. Sample characteristics are shown in Table 1. Participants were recruited through social media channels and online homepages of patient associations. A hyperlink at the recruitment notice brought them to the information letter, informed consent, and online survey. Participants did not receive a compensation for their participation.

**Table 1:** Characteristics of the participants in the online questionnaire (*N* = 686).

Gender, *n* (%)	
Male	45 (6.6%)
Female	641 (93.4%)
Age in years, mean (*SD*)	45.4 (11.8)
Nationality, *n* (%)	
Dutch	405 (59.0%)
Brazilian	114 (16.6%)
Belgian	50 (7.3%)
Greek	35 (5.1%)
Portuguese	29 (4.2%)
Peruvian	17 (2.5%)
Cypriot	12 (1.7%)
Other	35 (5.1%)
Relationship status, *n* (%)^a^	
Single	110 (16.0%)
In a relationship	71 (10.3%)
Married or cohabiting	444 (64.7%)
Separated or divorced	51 (7.4%)
Widowed	9 (1.3%)
Number of years of education after sixth birthday, *n* (%)	
0–9 years	34 (5.0%)
10–14 years	281 (41.0%)
15–19 years	283 (41.3%)
≥20 years	88 (12.8%)
Illness, *n* (%)^b^	
Fibromyalgia	303 (44.2%)
Irritable bowel syndrome	263 (38.3%)
Chronic fatigue syndrome	160 (23.3%)
Osteoarthritis	152 (22.2%)
Rheumatoid arthritis	139 (20.3%)
Psychiatric disorder	196 (28.6%)
Migraine	155 (22.6%)
Lung disease	118 (17.2%)
Cardiovascular disease (including high blood pressure)	90 (13.1%)
Job burnout	77 (11.2%)
Chronic skin condition	70 (10.2%)
Addiction	55 (8.0%)
Spondyloarthritis / Becherew's disease	51 (7.4%)
Diabetes	46 (6.7%)
Severe obesity	43 (6.3%)
Main illness diagnosed by, *n* (%)^c^	
Medical specialist	560 (81.6%)
Family physician	73 (10.6%)
Other health professional	33 (4.8%)
Self	10 (1.5%)
PHQ-15 score, mean (*SD*)	13.5 (5.0)

*Note*. PHQ-15: Patient Health Questionnaire-15.

a One missing case in the data.

b Only illnesses that occur in a minimum of 5% of the participants are shown.

c Ten missing cases in the data.

### Procedure

Threats and soothers were collected by an online survey that was created using LimeSurvey®. The survey was provided in Dutch, English, Greek, and (European and Brazilian) Portuguese. Participants were asked about threats and soothers they experienced. The following definition of threats was given to the participants: “threats create experiences of danger, harm, damage, or unsafety”. The given definition of soothers was: “comforts create feelings of calmness, well-being, safety, or social connectedness”. Using open-ended questions, participants were asked to mention as many as possible threats that may worsen their pain, fatigue, or other physical symptoms as well of comforts that may alleviate their pain, fatigue, or other physical symptoms. Answers in response to an additional open question about drives were not used in our current study, given that drives are considered to play a less clear role in worsening and alleviating physical symptoms (Pinto et al., 2023). The study was performed in compliance with the declaration of Helsinki and later amendments and approved by the Ethics Committee of the Faculty of Social and Behavioural Sciences of Utrecht University (FETC 19-219). Participation was anonymous and participants provided informed consent before participating. The data collection was done in October and November, 2019.

### Instruments

Participants provided the following demographic and clinical data: gender, age, nationality, relationship status, the number of years of education followed after their sixth birthday, whether and which rheumatic diseases and other illnesses (somatic and psychiatric) they had, and who diagnosed their main illness.

Participants also completed the Patient Health Questionnaire (PHQ-15; Kroenke et al., 2002). The PHQ-15 is a 15-item self-report questionnaire assessing somatic symptom severity within the past four weeks. Items are rated using a three-point Likert scale ranging from 0 (*not bothered at all*) to 2 (*bothered a lot*), and summed into a severity score that may range from 0 to 30. Scores of 5, 10, and 15 represent cutoffs for low, medium, and high somatic symptom severity, respectively. As suggested in a previous study (Kocalevent et al., 2013), missing values were replaced with the mean value of the remaining items in participants with no more than 20 percent of the items missing.

### Data selection

The extensive collection of threats and soothers was reduced to 40 threats and 40 soothers in several steps by a project group consisting of international master students in clinical psychology as part of their master's thesis under supervision of one member of the project group (RG; see Figure S1 in the supplementary file).

In a preparatory phase, the project group translated all non-English items to English. Single items containing more than one threat or soother (e.g., a threat item that included both “too little sleep” as well as “humid weather”) were split. As a preparatory step to support reduction of the abundant answers of participants, items that were more or less similar in content were loosely put together in columns in an Excel file.

In the first phase of the selection procedure during two consensus meetings, the project group selected representative sets of threats and soothers. Six criteria guided the selection. First, items that did not meet the definition of threats/soothers as provided in the online survey should be removed. Second, items that only applied to one group (e.g., only concerning one sex or age group) should be converted to items applying to more people. For instance, the threat items “menstrual complaints” or “pregnancy” were included in the more generic item “A physical symptom, for example, pain, fatigue, or stiffness”, and the soother item grandchildren was included in the item “Being surrounded by lovely people, for example, friends or family”. Third, items including examples of a specific threat or soother could be combined into one item (e.g., multiple items were included in the soother item: “a leisure activity, for example, reading, music, movies, dancing, drawing, painting, or another hobby”). Fourth, items consisting of conceptually distinct threats/soothers should be split into separate Items. Fifth, if the wording of the items was too ambiguous, abstract, or specific, the wording had to be changed; however, the wording should be as close to verbatim as possible. Sixth, non-indicative words, such as “too” and “often” should be removed from the items. The numbers of threat and soothing items after this phase were respectively 96 and 97.

In the second phase of the selection procedure, the selected threats and soothers were further reduced. First, pairs of members of the project group made suggestions to remove specific threats and soothers that were close in meaning so that varied sets were preserved. Subsequently, members of the project group individually and independently compared these suggestions made by pairs of members of the project group with the sets of 96 threats and 97 soothers that resulted after the first phase of the selection procedure. Their suggested amendments about which items should not be removed (because they reflected different content compared to the other items) or should be removed (because the items showed too much overlap with other threats and soothers) were discussed in a meeting until consensus was reached. The numbers of threat and soothing items after this phase were respectively 43 and 48.

The aim of the third phase of the selection procedure was to create manageable sets by reducing the number of resulting items. To that aim, all members of the project group independently sorted the threats and soothers, respectively, on similarity of content. When items were put on the same pile by each member of the project group and the project group agreed that there was considerable content overlap, then one or more items were either removed (as it had no differentiating value) or merged into a single item. After this phase, final sets of 40 threat and 40 soothing items were left.

## Study 2: card sorting

### Participants

Eligible participants were aged eighteen years or older and had an RMD or CSS diagnosis. Participants were recruited through online homepages and social media sites of national patient associations in the Netherlands. Those interested in getting more information received an informed consent and instruction booklet with card sorting materials and a reply envelope at home. These materials were sent to 316 Dutch participants. A sample size of 10 to 20 participants has been shown to be a workable number for concept mapping, ensuring a variety of opinions (Trochim, 1989). A resampling study involving 168 persons indicated that a sample size between 20 and 30 is an adequate choice (Wood & Wood, 2008). Our own experience with this method indicates that a sample size with a minimum of 50 is a safer choice, especially with varied materials that have to be sorted. Participants did not receive a compensation for their participation.

### Procedure

To structure the 40 threats and 40 soothers identified in Study 1, participants sorted them according to similarity of meaning in two separate card sorting tasks: one for threats and one for soothers. Also drives identified in Study 1 were sorted in a third card sorting task, but these were not analyzed in the current study. For each card sorting task, we provided the participants three sets of 40 cards each containing 40 threat items, 40 soother items, and 40 drive items. The cards were numbered. Participants sorted these sets separately. Taking into account that participants might want to stop after having done the sorting of one or two sets of cards, the order of the three card sorting tasks differed in the instruction booklets so that all three sets were equally likely to be sorted. In between each set, it was announced how much time it would take to sort the next set of cards and participants were given the opportunity to stop or go on.

To ascertain that the participants were thoughtful about their sorting and to prevent participants to make many or just a few groups of cards, in the accompanying instruction booklet, the following instruction was given to the participants with each set of cards: (1) all cards had to be grouped, (2) each card could be placed in only one group, (3) a minimum of four and a maximum of twelve groups had to be formed, and (4) each group could contain a minimum of two and a maximum of 20 cards. Participants were asked to report at answer forms which cards they grouped together by writing down the numbers of the items. Completed answer forms were sent back to the research group using an answering envelope.

The study was performed in compliance with the declaration of Helsinki and later amendments and approved by the Ethics Committee of the Faculty of Social and Behavioural Sciences of Utrecht University (FETC 19-274). Participation was anonymous and participants provided informed consent before participating. The card sorting was done from December 2019 to March 2020.

### Instruments

Participants provided the following demographic and clinical data: gender, age, relationship status, highest completed education level, rheumatic diseases and other illnesses (somatic and psychiatric), who diagnosed their main illness, and the number of years of having a diagnosis. Participants also completed the PHQ-15 (Kroenke et al., 2002).

### Data analysis

Before analysis, the sorted data were inspected. This was done because cluster analysis can only deal with complete sets of items being sorted. When a participant had not sorted three or fewer items or had sorted three or fewer of the same item in more than one group, these separate items were allocated to groups with a single item. A participant was removed from the sample when four or more items had not been sorted or when four or more items had been sorted in more than one group.

Hierarchical cluster analysis in SPSS version 25 (IBM Corp., Armonk, NY) was used to classify the threats and soothers that were individually sorted by the participants according to similarity of meaning. In cluster analysis, the cells of the input matrix of cards comprise the number of times that two cards were not sorted in the same pile. Squared Euclidean distances were computed between each pair of cards and Ward's method was used to derive the hierarchical structure of the sorts. This resulted in dendrograms of threats and soothers and corresponding agglomeration schedules showing which statements were being combined at each stage of the hierarchical cluster process. The number of clusters was decided by the project group and six patient research partners with an RMD or CSS based on meaning guided by visual inspection of distances in the dendrograms and the similarity and diversity of the threats and soothers included in the clusters. First, two members of the research team (KH, RG) decided on the initial cluster solution. Subsequently, four online subgroup meetings with patient research partners took place in which contents of both a lower and a higher number of clusters as determined by the agglomeration schedule were compared. Patient research partners were encouraged to decide on the preferred number of clusters as well as on cluster labels that best represented the included items. After this, a preliminary draft of clusters and cluster labels as well as contents of both a lower and a higher number of clusters were discussed with the other research members and the involved patient research partners, who were asked whether they agreed or disagreed with the chosen solution of clusters and corresponding labels. A final decision was taken on the basis of the feedback provided.

## Results

### Demographic and clinical characteristics

In Study 1, 686 participants completed the online survey yielding 2484 threats and 2329 soothing influences. These were summarized in sets of 40 threats and 40 soothers. Participants’ characteristics are shown in Table 1. A flowchart of the selection process of threats and soothers (Figure S1) is included in the supplementary file.

In Study 2, 118 participants completed the card sorting task for threats and/or soothers. Three participants who did not complete the card sorting task as instructed were removed from the sample, resulting in a sample of 115 participants (Table 2). Of these participants, one participant sorted soothers but not threats, four participants sorted threats but not soothers, and one participant sorted threats but was not included in the analysis of soothers because there were too many missing values. The majority of the sample was female (97.4 %) and married, in a registered partnership, or cohabiting (72.2 %). Most participants (85.2 %) had at least one CSS and 53 participants (46.1 %) reported at least one RMD other than fibromyalgia. Participants’ diagnoses were most often made by a medical specialist (93.9 %). The PHQ-15 score was minimal for one participant (.9 %), low for 25 participants (21.7 %), medium for 44 participants (38.3 %), high for 42 participants (36.5 %), and missing for three participants (2.6 %).

**Table 2:** Characteristics of the participants (*N* = 115).

Gender, *n* (%)	
Male	3 (2.6%)
Female	112 (97.4%)
Age in years, mean (*SD*)	48.2 (11.6)
Relationship status, *n* (%)	
Single	20 (17.4%)
In a relationship	5 (4.3%)
Married, registered partnership, or cohabiting	83 (72.2%)
Divorced or not cohabiting anymore	7 (6.1%)
Highest completed education level, *n* (%)	
Primary school	2 (1.7%)
Lower vocational education	7 (6.1%)
Advanced vocational and general secondary education	43 (37.4%)
Higher general secondary education	16 (13.9%)
University of applied sciences	35 (30.4%)
University	12 (10.4%)
Diagnosis, *n* (%)^a^^,^^b^	
Central sensitivity syndrome	98 (85.2%)
Rheumatic and musculoskeletal disease other than fibromyalgia	53 (46.1%)
Fibromyalgia	77 (67.0%)
Irritable bowel syndrome	62 (53.9%)
Chronic fatigue syndrome / Myalgic encephalomyelitis	16 (13.9%)
Osteoarthritis	39 (33.9%)
Rheumatoid arthritis	11 (9.6%)
Sjögren's syndrome	10 (8.7%)
Other rheumatic and musculoskeletal disease	14 (12.2%)
Comorbid diagnosis, *n* (%)^a^	
Lung disease	19 (16.5%)
Psychiatric illness	17 (14.8%)
Severe overweight	16 (13.9%)
Migraine	15 (13.0%)
Chronic skin condition	12 (10.4%)
Burnout	10 (8.7%)
Cardiovascular disease (including high blood pressure)	9 (7.8%)
Diabetes	7 (6.1%)
Main illness diagnosed by, *n* (%)	
Medical specialist	108 (93.9%)
Family physician	6 (5.2%)
Other health care professional	1 (.9%)
Self	0 (.0%)
Years of having a diagnosis, mean (*SD*)^b^	11.6 (11.4)
PHQ-15 score, mean (*SD*)^c^	12.9 (4.3)

*Note*. PHQ-15: Patient Health Questionnaire-15.

a Only illnesses that occur in a minimum of 5% of the participants are shown.

b One missing in the data.

c Three missings in the data.

### Threats

**Fig. 1:**
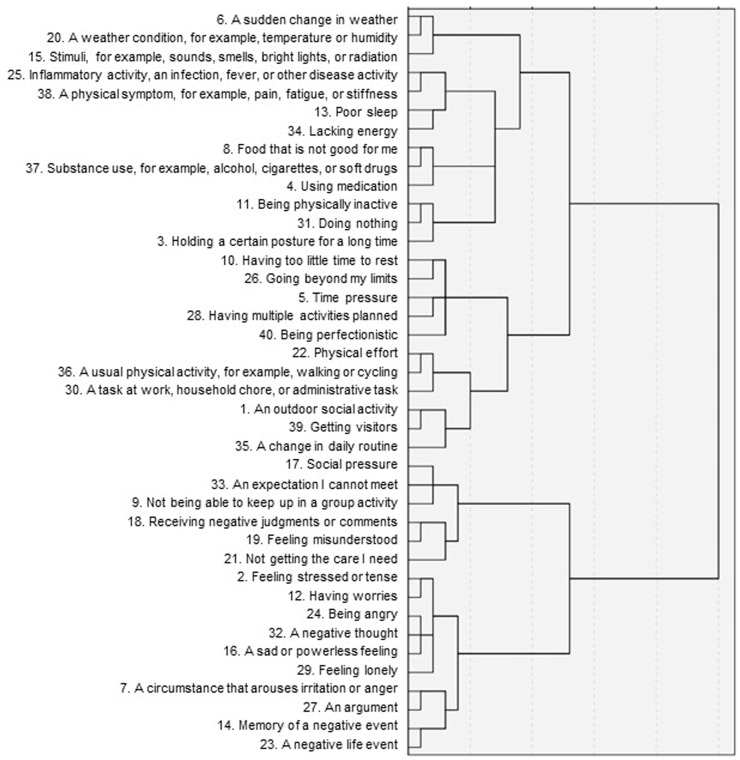
Dendrogram showing the structure of 40 threats.

The dendrogram of the hierarchical cluster analysis of threats is shown in Fig. 1. The items included within each cluster are also shown in Table 3. An eight-cluster solution was chosen: (1) environmental stimuli, (2) physical symptoms, (3) food and drugs, (4) inactivity, (5) demands, (6) effort, (7) invalidation, and (8) emotional stress. When deciding on the number of clusters, a seven- and a nine-cluster solution were also considered. Decreasing the number of clusters from eight to seven clusters would combine the clusters of food and drugs with inactivity. The project group decided not to combine these clusters. Although both clusters relate to lifestyle, they reflect a different content, may occur independently, and require different management. Increasing the number of clusters from eight to nine clusters would split the cluster of effort into two clusters (threat items 22, 36, and 30 vs. 1, 39, and 35, see Table 3). The project group decided not to split this cluster on the grounds that no clear distinction between those clusters was apparent (with both including items pertaining to effort).

**Table 3:** Overview of 40 threats and corresponding eight clusters.

**Cluster 1: Environmental stimuli** 6. A sudden change in weather 20. A weather condition, for example, temperature or humidity 15. Stimuli, for example, sounds, smells, bright lights, or radiation **Cluster 2: Physical symptoms** 25. Inflammatory activity, an infection, fever, or other disease activity 38. A physical symptom, for example, pain, fatigue, or stiffness 13. Poor sleep 34. Lacking energy **Cluster 3: Food and drugs** 8. Food that is not good for me 37. Substance use, for example, alcohol, cigarettes, or soft drugs 4. Using medication **Cluster 4: Inactivity** 11. Being physically inactive 31. Doing nothing 3. Holding a certain posture for a long time **Cluster 5: Demands** 10. Having too little time to rest 26. Going beyond my limits 5. Time pressure 28. Having multiple activities planned 40. Being perfectionistic	**Cluster 6: Effort** 22. Physical effort 36. A usual physical activity, for example, walking or cycling 30. A task at work, household chore, or administrative task 1. An outdoor social activity 39. Getting visitors 35. A change in daily routine **Cluster 7: Invalidation** 17. Social pressure 33. An expectation I cannot meet 9. Not being able to keep up in a group activity 18. Receiving negative judgments or comments 19. Feeling misunderstood 21. Not getting the care I need **Cluster 8: Emotional stress** 2. Feeling stressed or tense 12. Having worries 24. Being angry 32. A negative thought 16. A sad or powerless feeling 29. Feeling lonely 7. A circumstance that arouses irritation or anger 27. An argument 14. Memory of a negative event 23. A negative life event

*Note*. Threats ended with the sentence “… is a threat that may create an experience of danger, harm, damage, or unsafety.”

### Soothers

**Fig. 2:**
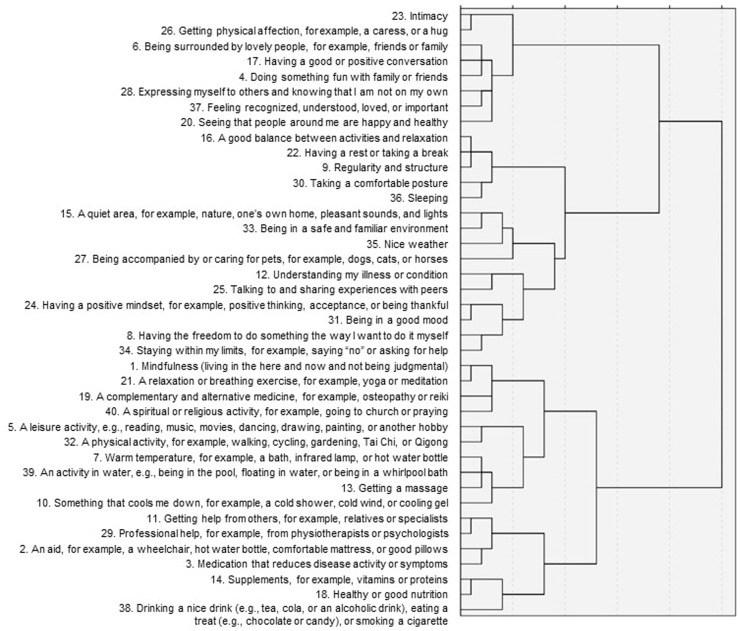
Dendrogram showing the structure of 40 soothers.

The dendrogram of the hierarchical cluster analysis of soothers is shown in Fig. 2. The items included within each cluster are also shown in Table 4. A ten-cluster solution was chosen: (1) social emotional support, (2) rest and balance, (3) pleasant surroundings, (4) illness understanding, (5) positive mindset and autonomy, (6) spirituality, (7) leisure activity, (8) wellness, (9) treatment and care, and (10) nutrition and treats. When deciding on the number of clusters, a nine- and an eleven-cluster solution were also considered. Decreasing the number of clusters from ten to nine clusters would combine the clusters of illness understanding with positive mindset and autonomy. The project group decided not to combine these clusters on the grounds that these are too different in content. Increasing the number of clusters from ten to eleven clusters would split the cluster of social emotional support into two clusters (soother items 23 and 26 vs. 6, 17, 4, 28, 37, and 20). Although one cluster is more physical and refers to more intimate encounters with close others, the project group decided not to split the cluster given that all items refer to social emotional contact.

**Table 4:** Overview of 40 soothers and corresponding ten clusters.

**Cluster 1: Social emotional support** 23. Intimacy 26. Getting physical affection, for example, a caress, or a hug 6. Being surrounded by lovely people, for example, friends or family 17. Having a good or positive conversation 4. Doing something fun with family or friends 28. Expressing myself to others and knowing that I am not on my own 37. Feeling recognized, understood, loved, or important 20. Seeing that people around me are happy and healthy **Cluster 2: Rest and balance** 16. A good balance between activities and relaxation 22. Having a rest or taking a break 9. Regularity and structure 30. Taking a comfortable posture 36. Sleeping **Cluster 3: Pleasant surroundings** 15. A quiet area, for example, nature, one's own home, pleasant sounds, and lights 33. Being in a safe and familiar environment 35. Nice weather 27. Being accompanied by or caring for pets, for example, dogs, cats, or horses **Cluster 4: Illness understanding** 12. Understanding my illness or condition 25. Talking to and sharing experiences with peers **Cluster 5: Positive mindset and autonomy** 24. Having a positive mindset, for example, positive thinking, acceptance, or being thankful 31. Being in a good mood 8. Having the freedom to do something the way I want to do it myself 34. Staying within my limits, for example, saying “no” or asking for help	**Cluster 6: Spirituality** 1. Mindfulness (living in the here and now and not being judgmental) 21. A relaxation or breathing exercise, for example, yoga or meditation 19. A complementary and alternative medicine, for example, osteopathy or reiki 40. A spiritual or religious activity, for example, going to church or praying **Cluster 7: Leisure activity** 5. A leisure activity, for example, reading, music, movies, dancing, drawing, painting, or another hobby 32. A physical activity, for example, walking, cycling, gardening, Tai Chi, or Qigong **Cluster 8: Wellness** 7. Warm temperature, for example, a bath, infrared lamp, or hot water bottle 39. An activity in water, for example, being in the pool, floating in water, or being in a whirlpool bath 13. Getting a massage 10. Something that cools me down, for example, a cold shower, cold wind, or cooling gel **Cluster 9: Treatment and care** 11. Getting help from others, for example, relatives or specialists 29. Professional help, for example, from physiotherapists or psychologists 2. An aid, for example, a wheelchair, hot water bottle, comfortable mattress, or good pillows 3. Medication that reduces disease activity or symptoms **Cluster 10: Nutrition and treats** 14. Supplements, for example, vitamins or proteins 18. Healthy or good nutrition 38. Drinking a nice drink (e.g., tea, cola, or an alcoholic drink), eating a treat (e.g., chocolate or candy), or smoking a cigarette

*Note.* Soothers ended with the sentence “… is a comfort that may create a feeling of calmness, well-being, safety, or social connectedness.”

## Discussion

This concept mapping study identified structured and encompassing overviews of threats and soothers regarding physical symptoms for people with RMD and CSS. Forty threats and 40 soothers were sorted by people with an RMD or CSS. This resulted in eight clusters of threats and ten of soothers. Threats were classified into clusters of “environmental stimuli”, “physical symptoms”, “food and drugs”, “inactivity”, “demands”, “effort”, “invalidation”, and “emotional stress”. Soothers were classified into clusters of “social emotional support”, “rest and balance”, “pleasant surroundings”, “illness understanding”, “positive mindset and autonomy”, “spirituality”, “leisure activity”, “wellness”, “treatment and care”, and “nutrition and treats”.

Several of the identified clusters of threats and soothers likely also apply to other groups than those of people with RMD and CSS; for example, threats such as “demands” (e.g., being perfectionistic; Limburg et al., 2017) and “emotional stress” (e.g., Hassett & Clauw, 2011) as well as soothing influences such as “social emotional support” (e.g., Harandi et al., 2017) and a “positive mindset” (e.g., MacLoad & Moore, 2000) are related to symptom severity in a variety of chronic illnesses. However, six of the eight threat clusters include physical aspects, which is likely an overrepresentation as compared to populations in which physical symptoms are less prominent. The taxonomy of soothers also includes some clusters that are likely more prevalent in people with chronic physical symptoms than in other populations, for example, the clusters “rest and balance” and “treatment and care”. Although the arrangement of threats and soothers may differ somewhat among subgroups, the taxonomy is based on a broad database of threats and soothers and may be used to uncover threats and soothers in individuals with an RMD or CSS.

This taxonomy could be used as a tool to assist in research and clinical practice. In research, it could be used to determine the occurrence and valence of threat and soothing clusters in stimulus materials, such as movies, pictures, or vignettes. This information could be used in experimental research, to examine, for instance, psychophysiological responses to stimulus materials with distinct clusters of threats and soothers. In clinical practice, the clusters may be part of a screening list to determine the occurrence of individual threat or soothing influences in patients. Such information can be used in shared decision-making to set up treatment goals focused on targeting threats or reinforcing soothers. Threat stimuli may, for instance, be targeted by cognitive-behavioral interventions such as systematic desensitization or cognitive restructuring. Soothing influences, in turn, may be reinforced by third-wave cognitive-behavioral therapies, such as acceptance and compassion-based therapies, which have shown promising results (Austin et al., 2021; Veehof et al., 2016). Dependent on the existence of and the type of imbalance between a hyperactive threat system and a hypoactive soothing system in a specific person, a focus on one of these systems or both may be needed to enhance treatment effects.

The threats and soothers in this study were semantically structured, that is, according to *meaning* and not according to *consistency of individual differences*. Therefore, this resulting taxonomy cannot be used to quantify threats or soothers with the aim, for instance, to compare groups. However, in an intended study, the 40 threat and 40 soothers items will serve as a basis to develop a questionnaire in which participants individually rate the strength of threats and soothers. Correlational approaches, for instance, factor or network analyses, could be used to structure items according to consistency of individual differences in order to derive reliable latent threat and soothing factors. With the newly developed instrument, the prevalence and strength of threats and soothers in individuals and groups could be determined. This would make it possible to examine the association of threat and soothing factors with their assumed neurobiological substrates, whether a persistent change in individual threat and soothing systems is possible following therapy, and whether such changes influence the nature and dynamics of the relationships between circumstances in an individual's life and the severity of physical symptoms.

Strengths of the study are the large number of people with an RMD or CSS that were included and that they were involved in several phases of the concept mapping procedure, such as reporting personal threats and soothers in the data collection phase, structuring the data in the sorting phase, determining the optimal number of clusters, and labelling of the clusters. However, also some limitations should be discussed. First, the threats and soothers were derived from a large sample, including—besides people with an RMD or CSS—also other people with chronic physical symptoms. Although this guaranteed a comprehensive set of threats and soothers, it is possible that some of the threats and soothers are less relevant to people with an RMD or CSS. Second, the reduction of thousands of threats and soothers to manageable sets was more semantic than phenomenological, because it was done by students instead of people with chronic physical symptoms. Third, the diagnoses of the participants were based on self-report without certification of the diagnosis by a medical specialist. Nevertheless, virtually all respondents indicated that a physician or health professional diagnosed their main illness. Fourth, only few men were represented in both Studies 1 and 2. Therefore, items that are represented in the overviews may be more relevant to women, and we cannot be sure that the observed structure fully applies to men with RMD and CSS. A greater female prevalence is common in both CSS (e.g., Lim et al., 2020; Heidari et al., 2017; Lovell & Ford, 2012) and most RMD (e.g., Branco et al., 2016). The higher number of female participants may also be explained by women participating more often in online survey studies (e.g., Wu et al., 2022). Perhaps other factors play a role, such as gender disparities in members of patient associations that helped with recruitment of participants, or in social media that were predominantly used in this study (e.g., more Facebook than X). To address the gender imbalance in our samples, we took care that items were not gender-specific. Practical application of the taxonomy may show whether it is useful in clinical practice for men. Fifth, race and ethnicity were not measured, therefore, we do not know whether threats and soothers experienced by minorities are represented. Sixth, without additional research, the taxonomy cannot be used in groups other than people with an RMD or CSS.

To conclude, our study provides a structured and comprehensive overview of threat and soothing influences in people with an RMD or CSS. The results can be used as a taxonomy in experimental research to label threat and soothing stimuli and in clinical practice to screen and monitor relevant treatments targets.

## Funding

This research did not receive any specific grant from funding agencies in the public, commercial, or not-for-profit sectors.

## Data availability statement

Data are available upon reasonable request to the corresponding author.

## Declaration of Competing Interest

The authors declare that they have no known competing financial interests or personal relationships that could have appeared to influence the work reported in this paper.

## Appendix Supplementary materials

Image, application 1
