# Supplementary material for: A taxonomy of threat and soothing influences in rheumatic and musculoskeletal diseases and central sensitivity syndromes
Source: Int J Clin Health Psychol. 2023 Nov 23;24(1):100420. doi: 10.1016/j.ijchp.2023.100420 (PMC10701127; doi:10.1016/j.ijchp.2023.100420)
Supplement: Supplementary file 1 [file mmc1.docx]

**Supplementary file of the article “A Taxonomy of Threat and Soothing Influences in Rheumatic and Musculoskeletal Diseases and Central Sensitivity Syndromes”**

**Figure S1**

*Flowchart of the three phases of the selection procedure for threats and soothers*

*
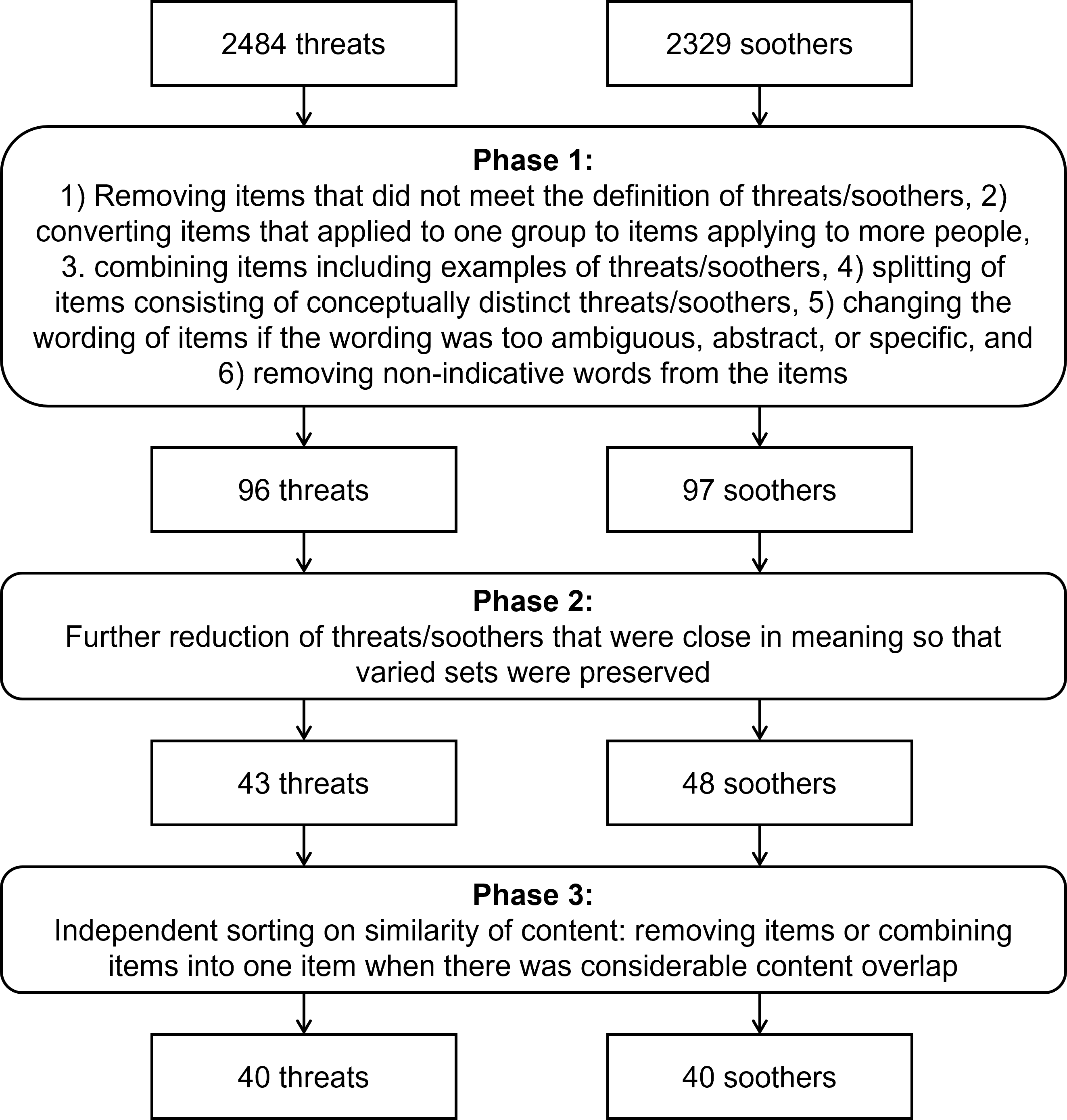
*
